# Co-designing Nova Sleepcare: exploring in-clinic, physician-delivered sleep education

**DOI:** 10.3389/fped.2026.1760205

**Published:** 2026-07-30

**Authors:** Ishnoor K. Nahal, Myka Estes, Genevieve Currie, Callum Heathcote, Greta Heathcote, Elizabeth Deliscar, Suzanne Deliscar, Megan Thomas, Carrah Bouma, Anamaria Richardson, Deborah Dewey, Sarah J. MacEachern

**Affiliations:** 1 Department of Pediatrics, Cumming School of Medicine, University of Calgary, Calgary, AB, Canada; 2 Alberta Children’s Hospital Research Institute, Cumming School of Medicine, University of Calgary, Calgary, AB, Canada; 3 School of Nursing and Midwifery, Mount Royal University, Calgary, Alberta, Canada; 4 Department of Pediatrics, Dalhousie University, Halifax, NS, Canada; 5 IWK Health, Halifax, NS, Canada; 6 Faculty of Health and Medicine, Lancaster University, Lancaster, United Kingdom; 7 Department of Pediatrics, Faculty of Medicine, University of British Columbia, Vancouver, BC, Canada; 8 BC Children's Hospital Research Institute, Vancouver, BC, Canada; 9 Department of Community Health Sciences, Cumming School of Medicine, University of Calgary, Calgary, Alberta, Canada; 10 Hotchkiss Brain Institute, University of Calgary, Calgary, Alberta, Canada

**Keywords:** behaviors of concern, neurodevelopmental disorders, reflexive thematic analysis, co-design, pediatric, sleep education

## Abstract

**Introduction:**

Youth with neurodevelopmental disorders (NDD) frequently experience behaviors of concern (BoC), such as aggression or self-injury, often exacerbated by sleep disturbances. Sleep education can improve sleep in pediatric populations, but existing interventions rarely fit into routine clinical care for families managing these behaviors. As such, effective programs often fail to reach those who need them the most.

**Objective:**

To co-design Nova Sleepcare, an in-clinic, physician-delivered sleep education program, by identifying barriers and facilitators to its use among end-users (youth with NDDs, caregivers, and pediatricians).

**Methods:**

A prototype of Nova Sleepcare, based on clinical guidelines and evidence-based behavioral strategies, was iteratively refined through open-ended interviews with 24 end-users (8 youth, 9 caregivers, 7 pediatricians). Interviews were transcribed verbatim and analyzed using reflexive thematic analysis to identify barriers, facilitators, and modifications needed to optimize Nova Sleepcare for real-world practice

**Results:**

Analysis identified one barrier and three key facilitators that shaped the final intervention. The barrier reflected end-users’ limited capacity to deliver or implement sleep strategies. Facilitators included: (1) strong motivation among youth and caregivers to improve sleep and reduce BoC; (2) Nova Sleepcare's accessibility and ability to be tailored across diverse presentations and settings; and (3) its stepwise Pathway to Better Sleep, helping families and pediatricians collaboratively prioritize manageable strategies. These findings informed a streamlined, flexible tool that end-users viewed as practical and relevant

**Conclusion:**

By embedding co-designed, evidence-based, active sleep education into the clinical encounter, Nova Sleepcare addresses a longstanding gap in care for youth with NDDs with BoC.

## Introduction

Improving the treatment of behaviors of concern (BoC) in youth diagnosed with neurodevelopmental disorders (NDDs, *e.g.,* autism, intellectual disability) is imperative. Many youth with NDDs, who comprise 8%–18% of children in developed countries, display these behaviors (1–7), which can include aggression (*e.g.,* punching, hair pulling), self-injury (*e.g.,* skin picking, eye gouging), and inappropriate conduct (*e.g.,* fecal smearing, unremitting screaming) (3, 8–11). These behaviors affect up to 80% of youth with NDDs, restricting access to education and community activities (9–12), reducing quality of life for families, and, in severe cases, leading to hospitalization or out-of-home placement (13–21).

Sleep disturbances, which include general insomnia-related symptoms (*e.g.,* difficulty initiating or maintaining sleep) and short sleep duration, are strongly linked to increased BoC, impact up to 86% of youth with NDDs (22–29). Studies show that improving sleep can reduce BoC and improve functioning (30–37), but access to effective, individualized sleep support remains limited. Sleep education, the collaborative provision of sleep-related information to improve sleep, is an evidence-based, first-line treatment delivered in formats such as multiweek caregiver training (34, 38), multi-hour workshops (31), or intensive, tailored sleep plans (39). These approaches are effective, but often inaccessible to families in crisis as they require time, capacity, or eligibility many families are unable to realize. Clinicians are left to refer families to external programs that may be burdensome, delayed by waitlists, or unavailable to children with complex presentations. Recognizing these accessibility barriers, researchers have explored delivering sleep education directly within routine clinic visits, though early attempts revealed the limitations of passive information sharing.

A randomized trial found that simply handing caregivers sleep information during clinic visits was no more effective than usual care (40). In follow-up interviews, caregivers emphasized a need for implementation support: tailored, practical guidance that fit their specific circumstances (40). Later versions of the program incorporated individualized support and improved sleep outcomes (41, 42), but like many otherwise effective pediatric sleep interventions, they could not be delivered within routine clinical workflows in primary pediatric care settings. These programs often require additional infrastructure and staffing, creating an “implementation cliff” (43) where efficacy does not translate into accessibility for families. The very features that make these interventions successful (intensity, customization, and specialized personnel) also make them difficult to spread and scale. Moreover, there is an overall lack of evidence-based behavioral sleep training and materials available to pediatric physicians (44). Without appropriate training or knowledge, physicians may not be equipped to evaluate and treat pediatric sleep disturbances.

Thus, there is a critical need for a brief, individualized sleep intervention that can be delivered at the point of care in primary pediatric care: namely, during physician visits when sleep and BoC issues are raised without requiring referral or additional appointments (45, 46). Specifically, To address this gap, we developed an initial prototype for embedding individualized, evidence-based, active sleep education directly into routine clinical encounters in primary pediatric care while accommodating diverse presentations common among youth with NDDs. Through an iterative co-design process with end users, this study aimed to: (1) identify facilitators and barriers to physician-delivered sleep education among youth, caregivers, and pediatricians, and (2) refine the prototype into Nova Sleepcare, a clinically feasible and family-acceptable intervention optimized for real-world implementation.

## Methods

### Study design

Co-design was conducted in partnership with youth, caregivers, and pediatricians to create an intervention that is inclusive to lived experience and is aligned with end-user needs (47, 48). We used qualitative description methodology to explore facilitators of and barriers to implementing physician-delivered sleep education in clinic. There was no *priori categorization* (*e.g.,* predefined theoretical framework, hypotheses, questions) that guided the social interaction between the research team and research partner(s) (49). As a result, we generated questions based on the narration the research partner provided (49). Through open-ended interviews rooted in naturalistic inquiry (50), we addressed the following research question: “What are the responses (attitudes, thoughts, and perspectives) of youth with NDDs, their caregivers, and pediatricians towards an initial draft of Nova Sleepcare?”

### Research partners and recruitment

Following ethics approval from the University of Calgary's Conjoint Health Research Ethics Board (REB22-1525) and Alberta Health Services, we recruited three end-user groups through purposive sampling. Youth research partners (aged 8–17) had a diagnosed NDD and co-occurring BoC and sleep problems or were siblings of youth meeting these criteria. Caregiver were eligible to participate if they were biological, adoptive, foster parents, or grandparents of eligible youth. Pediatricians (general and subspecialist pediatricians) were recruited as the specialists most likely to manage these complex presentations.

Youth and caregivers were recruited from local organizations and institutions that serve neurodiverse youth. Pediatric physicians were recruited through pre-existing working relationships, direct email communication, and physician mailing lists. Neurodiverse youth and caregivers were compensated $25/hour as per Canada's Strategy for Patient-Oriented Research. Physicians were compensated $100/hour per the Canadian Medical Association guidelines on physician remuneration.

### Nova Sleepcare materials

Nova Sleepcare was designed to deliver active sleep education: a collaborative process in which pediatricians work with families to discuss evidence-based sleep hygiene, behavioral strategies, and melatonin while co-developing practical, customized plans that fit the child's needs and family circumstances. This approach differs from passive information sharing by emphasizing physician-family collaboration and individualized problem-solving during the clinical encounter.

The initial Nova Sleepcare prototype integrated evidence-based sleep education content into clinic-deliverable formats. Materials included sample scripts, physician reference guides, youth and caregiver handouts, the ‘Pathway to Better Sleep’ goal-setting framework and a supporting website (http://www.novasleepcare.com). Content addressed sleep physiology, behavioral strategies, environmental modifications, and evidence-based melatonin protocols, derived from clinical practice guidelines for pediatric insomnia (51–54)

### Data collection

Research partners were informed about the purpose of the interview and examples of guiding questions during a follow-up email or phone call. These guiding questions focused on the impact of sleep disturbances, sleep strategies, and responses to the initial materials of Nova Sleepcare.

We conducted 16 open-ended interviews (60–90 min) either in-person or virtually. Research partners reviewed Nova Sleepcare materials before interviews, which were iteratively refined based on emerging feedback. This involved revising materials based on partner feedback prior to upcoming interviews. All sessions were recorded, transcribed verbatim, and managed using NVivo 14

### Data analysis

The analysis was led by a graduate student (IKN), an Indo-Canadian, cisgender woman, and a research manager (ME), a white, cisgender woman and experienced researcher with longstanding engagement in NDD research and clinical partnerships. Both authors approached this work as informed outsiders, drawing on extensive experience working with neurodiverse populations but not identifying as neurodiverse themselves. Prior to coding, IKN and ME immersed themselves in the data by transcribing audio recordings, re-listening to the audio recordings, and making casual notes while rereading transcripts. Next, IKN and ME coded transcripts by organizing data around similar meanings by using “clear labels” that tagged “chunks” of data (55). These codes were grouped together to provide insight into potential facilitators and barriers of the dataset (55). Throughout the coding process, IKN and ME met weekly to engage in “critical friend discussions” to ask provocative questions and offer critiques to provide alternative perspectives to the dataset (56). Lastly, IKN and ME compared thematic frameworks and reanalyzed the connections among the codes, subthemes, and themes to construct a logical argument of the dataset.

The finalized thematic framework was reviewed, triangulated, and confirmed for plausibility by the senior author (SJM) and her lab's Advisory Council, which consists of two caregiver-youth dyads who have lived and practical experience. The Advisory Council participated as research partners in the open-ended interviews, provided additional feedback via three hybrid sessions, shared in decision-making regarding the content and physical design of intervention materials, and were compensated $30/hour for their involvement. All Advisory Council members join this work as co-authors. CH is a pre-teen boy with a neurodevelopmental disorder and GH is CH's mother. ED is a teenage girl with a rare genetic disorder and two neurodevelopmental disorders and SD is ED's mother.

Rigor of the qualitative analyses were ensured through investigator authority, engagement with a diverse range of research partners, and the creation of detailed descriptions of our study design process and analysis informed by a systematic search of existing literature. IKN kept an audit trail to track changes to study protocols and concerns or comments identified by research partners and the study team.

## Results

Thematic saturation was achieved after conducting 16 open-ended interviews with 24 research partners: 8 youth, 9 caregivers, and 7 pediatricians, as no new insights were generated (57). Research partner demographics are presented in Tables 1–3.

**Table 1:** Characteristics of caregivers.

Caregiver Demographics	*n* = 9 (%)
**Age**	
18–24	1 (11%)
35–39	2 (22%)
40–44	2 (22%)
45–49	2 (22%)
50–54	1 (11%)
No Response	1 (11%)
**Relationship to Youth Partner**	
Biological Mother	6 (67%)
Biological Father	2 (22%)
Sibling	1 (11%)
**Relationship Status**	
Married or Common-Law	5 (56%)
Divorced or Separated	2 (22%)
No Response	2 (22%)
**Race**	
White	5 (56%)
South Asian	2 (22%)
Black	1 (11%)
No Response	1 (11%)
**Community of Residence**	
Large urban population, with a population of 100,000 or more	4 (44%)
Medium population centre, with a population between 30,000 and 99, 999	1 (11%)
Small population centre, with a population of 1,000 and 29,999	2 (22%)
No Response	2 (22%)
**Highest Level of Education**	
Undergraduate Degree/Bachelor's Degree	2 (22%)
Graduate Degree	4 (44%)
Diploma or Certificate	1 (11%)
No Response	2 (22%)
**Current Employment Status**	
Unemployed	1 (11%)
Part Time	1 (11%)
Full Time	4 (44%)
Homemaker	1 (11%)
No Response	2 (22%)
**Immigration Status**	
Canadian citizen by birth	6 (67%)
Canadian citizen by naturalization	1 (11%)

**Table 2:** Characteristics of youth.

Youth Demographics	*n* = 8 (%)
**Age**	
10	2 (25%)
11	2 (25%)
13	1 (12.5%)
14	1 (12.5%)
15	2 (25%)
**Sex**	
Female	2 (25%)
Male	5 (62.5%)
No Response	1 (12.5%)
**Gender**	
Man or Boy	5 (62.5%)
Woman or Girl	2 (25%)
Non-Binary	1 (12.5%)
**Race**	
White	5 (62.5%)
South Asian	1 (12.5%)
Black	1 (12.5%)
No Response	1 (12.5%)
**Number of Siblings**	
0	3 (37.5%)
1	3 (37.5%)
2	1 (12.5%)
No Response	1 (12.5%)
**Immigration Status**	
Canadian Citizen by Birth	6 (75%)
Canadian Citizen by Naturalization	1 (12.5%)
No Response	1 (12.5%)
**Neurodevelopmental Disorder Diagnosis** ^a^	
Intellectual Disability	1 (12.5%)
Attention Deficit Hyperactivity Disorder	5 (62.5%)
Specific Learning Disorder	3 (37.5%)
Motor Disorder	1 (12.5%)
Unspecified Neurodevelopmental Disorder	1 (12.5%)
Specific Genetic Disorder	1 (12.5%)
**Co-Occurring Conditions**	
Genetic Disorder	1 (12.5%)
Generalized Anxiety Disorder	1 (12.5%)

a Sum is greater than 100%.

**Table 3:** Characteristics of pediatricians.

Physician Demographics	*n* = 7 (%)
**Age**	
35 to 39	2 (28.5%)
40 to 44	1 (14.3%)
45 to 49	1 (14.3%)
50 to 54	1 (14.3%)
No Response	2 (28.5%)
**Years of Practicing Medicine**	
5–10	2 (28.5%)
11–15	2 (28.5%)
16–20	1 (14.3%)
No Response	2 (28.5%)
**Location of Practice**	
Large urban population, with a population of 100,000 or more	5 (71.4%)
No Response	2 (28.5%)
**Practice Setting**	
Academic Center (University based)	1 (14.3%)
Private Practice	1 (14.3%)
Hospital	1 (14.3%)
Community Health Centre	1 (14.3%)
Combination (Private Practice, Non-Profit, and Specialized Indigenous Health Clinic)	1 (14.3%)
No Response	2 (28.5%)
**Specialty**	
Pediatrician	3 (42.9%)
Developmental Pediatrician	1 (14.3%)
Neurologist	1 (14.3%)
No Response	2 (28.5%)
**Race**	
White	5 (71/4%)
No Response	2 (28.5%)
**Sex**	
Male	1 (14.3%)
Female	4 (57.3%)
No Response	2 (28.5%)
**Gender**	
Woman or girl	4 (57.3%)
Man or boy	1 (14.3%)
No Response	2 (28.5%)
**Immigration Status**	
Canadian citizen by birth	5 (71/4%)
No Response	2 (28.5%)

### Barriers and facilitators to implementation

**Figure 1:**
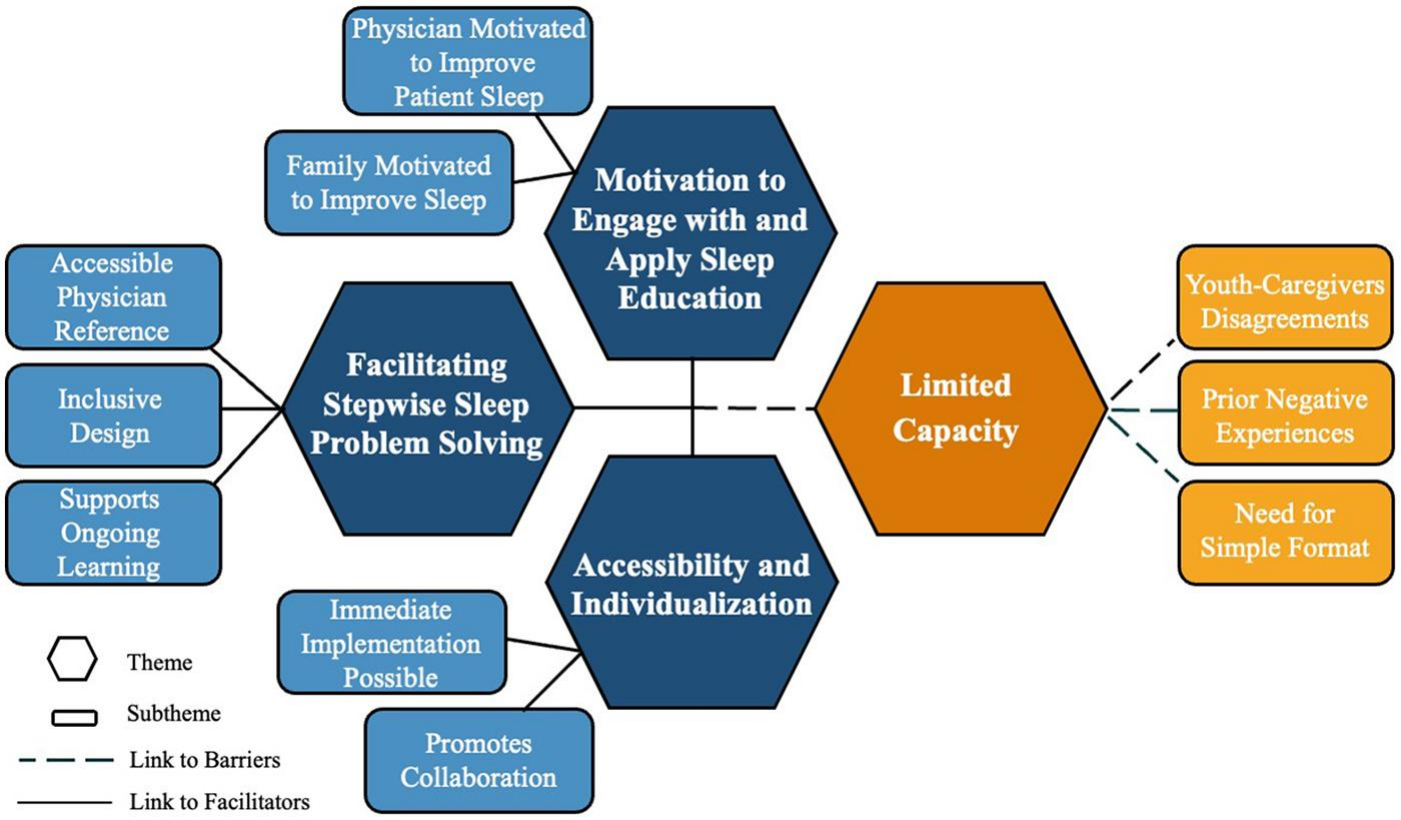
An overview of the thematic framework.

Analysis revealed four major themes representing barriers to and facilitators of implementing physician-delivered sleep education (Figure 1). We present these themes in order of their prominence across research partner narratives (Table 4).

**Table 4:** Thematic framework with exemplar quotations.

Theme	Exemplar Quotes
Limited Capacity as a Primary Implementation Barrier ***Subtheme*** Conflicting Perspectives Between Youth and Caregivers	*“I was up all night, and through all of this, he's not sleeping and I'm also not sleeping, and have two other kids and a job and I had a bit of a meltdown at [hospital], and was like, there has to be something else. We cannot go on like this.”* –Caregiver *“Normally when we wake* [sibling] *up, it's his signature mad face, which is hilarious. And then it's hard to not laugh and then gets mad when we laugh and normally that results in him punching us*” – Youth “*…it might be nice to have a graduated approach or a flow diagram. Like start by just implementing one strategy at a time for one week or for two weeks at a time and slowly building up on these recommendations and we recognize and how hard it is to implement change into your lifestyle.”* – Physician *“…from a user perspective, I don't think any Pediatricians* [are] *going to read this* [script] *and they’re going to take what they take from this, and they’re going to use their own script*” –Physician “*She should be asleep by maybe 9 or 9:30pm. This is what it should be for her*” – Caregiver “*But when I sleep, that isn't my bedtime…and I’m happy with it. I’m happy if I got at least 5 h of sleep*” – Youth “*There's a downtown at 8pm on my phone where it just stops working except for emergency phone calls. And no matter what time it is, if I’ve been on my phone for more than 4 h, it just shuts off and doesn't work anymore*. *I’m not sure if I agree with this because I like to have a way to fill up my time as well as space but* [my parents are] *doing it*” – Youth
Motivation to Engage with and Apply Sleep Education as a Facilitator	*“I think another thing would be to have information on when a good night's sleep takes effect…because by having to change your routine or maybe being disruptive then what does a goodnight sleep feel like*?” – Caregiver “*But I guess my thought was thinking about common things, like Sleep Association that may be lasting longer than the toddler phase and even into early childhood. May be something worth mentioning and approaches or behavioral strategies around it*.” – Physician
Accessibility and Individualization of Nova Sleepcare as a Facilitator	*“And so we've actually been at this, and I've been trying* [Nova Sleepcare since we] *read it over”* – Caregiver *“The thing is after reading the document *[Nova Sleepcare]* nowadays, she's not watching any screens before going to bed. She's following that.” –* Caregiver “*You wrote this *[Nova Sleepcare]* at a grade five level for parents, which I think is good for parents” –* Caregiver *“…he enjoyed looking at *[Nova Sleepcare]*, and he engaged when we talked about it, so that is a good a sign as any that he was good with it” –* Caregiver “*I liked how* [the handouts] *pointed out what was good and what wasn't*…*and I liked that there were bullet points and had the different sections”* – Youth “*That's how you engage and make sure they have an understanding and are listening to you and it gives them a say, so it makes it more of a shared conversation rather than, I'm telling you this, congress lecture style”* – Physician
Facilitating Stepwise Sleep Problem Solving Through Nova Sleepcare	*“So, I would say I will use my motivational interviewing conversational skills to encourage residents to feel familiar. And obviously we can lead by example, by showing* [residents]…*and they can see it being done in action.”* – Physician “*If the* [sleep specialist] *had gone through it this way with us, it would have changed how I felt about the visit completely*” *–* Caregiver *“I liked the choose your own adventure type thing where this would be more applicable for me, and I think it really depends on the family and what their capacity is to engage, and I also think how that information is presented*” *–* Caregiver

### Theme 1: limited capacity as a primary implementation barrier

The most pervasive barrier identified across all research partner groups was end users’ limited resources and capacity to deliver or implement sleep strategies. When asked about their experiences addressing sleep problems, all nine caregivers described overwhelming challenges. These included managing chronic and severe sleep disturbances (9/9), family dysfunction (7/9), supporting children in distress (4/9), addressing detrimental impacts on daytime function (3/9), difficulties disengaging youth from screens (2/9), and unsatisfying specialist encounters (3/9). For example, a caregiver reflected:

“…I think if I’m tired, and I’m exhausted, and [with what] they say about complex needs, and all of these things are coming at me, and on top of that sleep hygiene stuff that I’m probably not even doing myself as parents, by the time I’m ready to go to sleep, I need a hour to myself”.

Pediatricians expressed concerns that existing sleep resources, while potentially effective, were impractical for families facing a multitude of constraints (5/7). One pediatrician shared:

“…the problem with the other resources is that it sounds great to see a psychologist, it sounds great to do all these programs, but the limiting factor is time and money…I have a ton of foster kids who do not have the resources…”.

Four pediatricians also noted long waitlists for sleep specialists and emphasized that these providers often lack expertise in supporting the needs of youth with NDDs with complex presentations.

Pediatricians and caregivers emphasized the need for a simple, actionable format for sleep problem solving that accommodates limited time and capacity. Four pediatricians found the initial Nova Sleepcare scripts too cumbersome:

“…the sample script is a lot to go through”

“…it takes a lot of work. And I don't know if pediatricians have the time to put in the work to get all of it.”

Six pediatricians recommended a more streamlined format, prompting the development of a concise physician-facing handout. Pediatricians also raised concerns about caregiver capacity: two questioned whether families could realistically implement recommended sleep strategies at home, regardless of resource quality. In response, three pediatricians suggested a graduated, stepwise format, such as a flow diagram, to help families build changes incrementally. Two caregivers independently recommended that materials be delivered in small, digestible pieces. In response, we developed the ‘Pathway to Better Sleep’, a goal-setting framework that structures Nova Sleepcare content into focused, achievable steps.

### Subtheme: Conflicting perspectives between youth and caregivers

Although youth and caregivers agreed that improving sleep was important, they frequently disagreed on both the causes of sleep problems and the acceptability of specific strategies. In all six youth–caregiver dyads, tensions emerged around interventions such as weighted blankets, melatonin, or sleep fading; four also disagreed on the role of technology. These disagreements often compounded families’ sense of being overwhelmed, making it harder to act on recommendations and highlighting the need for approaches that mediate between perspectives. One youth reflected on the strain of trying to show progress to their parents:

“…the whole thing happening [with bedtime fading] made me feel like I had to show results. So, I was just trying harder to stay in bed and be normal and regular. So, sometimes even when I was tired, I didn't talk about it so that [my parents] would think it was getting better…”

### Theme 2: Motivation to engage with and apply sleep education as a facilitator

Youth and caregivers expressed high motivation to address the connection between sleep and behavior, actively seeking strategies beyond those provided in the Nova Sleepcare prototype. They identified gaps and proposed additional topics and tools that would help improve sleep. Four youth and five caregivers mentioned blackout shades, sound machines, laying down with a service dog, listening to mellow music, singing, playing with Legos, mindfulness practice, drawing, and having a caregiver present. These examples reflected their active interest in finding personalized ways to manage sleep problems.

Youth also demonstrated a nuanced understanding of how sleep problems affected their lives, often extending beyond simple fatigue. Six research partners described impacts on emotional well-being, social functioning, and family and school relationships, linking poor sleep to heightened anxiety and fear, irritability, sadness or depression, and feeling pressure to act “normal.” One youth shared:

“I get a lot of worries when I don't get good sleep…I could worry about, I don't know, homework, which I already finished. Basically, anything that could go wrong I worry about”.

These perspectives underscore the importance of engaging youth directly in problem-solving, as their lived experience can guide more sustainable behavior change.

As Nova Sleepcare incorporated research partner feedback, this motivation strengthened. Caregivers reported the revised materials as more accessible (4/9), comprehensive (5/9), easy to understand (8/9), and essential for effective sleep problem-solving (7/9). Similarly, six youth noted that the final version felt more relatable (4/6) and visually appealing (5/6), reinforcing the value of tailoring the intervention to reflect their lived experience and preferences.

Pediatricians expressed progressively greater confidence in Nova Sleepcare as successive iterations incorporated their feedback. Pediatricians who reviewed the initial draft raised concerns about complexity and time burden, noting that the materials felt cumbersome for routine visits. These concerns were addressed through streamlining and simplification, resulting in later versions that fit more naturally into clinical workflows. Pediatricians presented with the refined versions responded more positively, reporting that the materials felt practical and clinically relevant, and by the final iteration, no additional major changes were requested. Along the way, pediatricians also offered targeted suggestions to enhance the content's utility, such as distinguishing between daytime and nighttime calming strategies (2/7), supporting caregiver modeling of screen limits (2/7), explaining sleep onset associations (1/7), and contrasting melatonin with over-the-counter alternatives like Benadryl (1/7). They emphasized the importance of using approachable language (4/7) and simplifying complex topics such as melatonin physiology and dosing (3/7), feedback that further informed the final version.

### Theme 3: Accessibility and individualization of Nova sleepcare as a facilitator

Pediatricians emphasized that Nova Sleepcare fills a critical gap in current practice: it provides high-quality, individualized guidance that can be tailored to the physician's clinical style, patient population, and family context. All seven pediatricians viewed Nova Sleepcare as a practical tool to improve care, describing its content as “…*nice, simple,* [and] *straightforward.*” They highlighted its relevance across diagnostic categories and levels of complexity, noting that few existing resources accommodate the co-occurring medical, psychiatric, and developmental conditions seen in their practices. As one pediatrician shared:

“…in addition to teenagers with other medical issues, [such as] substance abuse and psychiatric stuff, I think this is generalizable to both, and I think the way that we would actually talk about it would depend on the population.”

Pediatricians also valued Nova Sleepcare for aligning with clinical guidelines (6/7), supporting behavior change (5/7), improving family functioning (6/7), and promoting youth well-being (5/7). They saw it as a resource that families could revisit after the clinical consultation, reinforcing clinical recommendations. One pediatrician noted:

“…often you come out into the community, and you don't know where to access resources…and so this would be a beautiful tool to have in your back pocket”.

Another emphasized its value for overwhelmed families:

“…you can only take away so much as a parent and [they are] already overwhelmed and so having something to refer back to afterwards…you can actually consolidate it and say ‘oh yeah, these were the specific things that we talked about’”.

Youth and caregivers similarly described Nova Sleepcare as inclusive, relevant, and adaptable. Five youth found the visuals digestible and teen-appropriate, reinforcing the importance of engaging design. As one caregiver observed:

“…our kids do like to see themselves represented in things… that might be another way to engage these kids in this content.”

Another added:

“…this looks very good, because it illustrates how sleep can be hard work and how can we make sleep easier… this would be really good for social stories for children on the autism spectrum.”

The Nova Sleepcare website was frequently cited as an accessible, trusted platform that extends the program's impact beyond the initial clinic visit. Caregivers appreciated its ability to provide deeper dives into behavioral strategies (3/8) and to correct common misconceptions (4/8). One caregiver reflected:

“…if I’m up at 3am and I’ve got to scroll through something on my phone to stay away from my child who is still going back to sleep, it's a place I can go and get some further information.”

Both caregivers and youth described the website as reinforcing and expanding on what was discussed during clinic visits.

### Theme 4: Facilitating stepwise sleep problem solving through Nova sleepcare

End-user groups described the ‘Pathway to Better Sleep’—Nova Sleepcare's priority-setting framework—as a pragmatic tool because its strategies could be tailored to each family's needs. One pediatrician highlighted how the framework helped avoid overwhelming caregivers: “…*look at this caregiver handout and feel like you have to try it all, because that's not reasonable*” but rather “…*pick and choose what strategies you feel is best for your family…”*.

One youth stated that they “*loved the step-by-step instructions*” while five caregivers disclosed positive feelings to the ‘Pathway to Better Sleep’, characterizing this component as accessible (3/5), family-centric (2/5), and individualizable (3/5).

Three caregivers found the in-clinic, physician-delivered aspect of Nova Sleepcare necessary to prioritize collaboration among end-users. One caregiver shared how the youth handout could encourage their son's pediatrician to “…*specifically* [ask] *him questions in a way that* [is] *very useful for him and that he could understand*”.

## Discussion

This study explored barriers and facilitators to Nova Sleepcare, an in-clinic, physician-delivered sleep education tool for youth with NDDs with BoC and their caregivers. Across interviews, end-users emphasized the need for timely, individualized support that fits within real-world clinical practice. Families expressed high motivation to address sleep problems yet described prior experiences with rigid or burdensome programs that taxed already limited resources. These findings reinforce the need for accessible, brief, and flexible interventions for sleep that can be delivered during physician visits for youth and families seeking help to decrease BoC.

End-users valued Nova Sleepcare not only as a source of practical knowledge but also as a tool to scaffold collaborative problem-solving through shared goal setting. While goal setting is a well-established strategy for promoting behavior change across complex and chronic health conditions (58–61), its effectiveness depends on how and with whom those goals are set. Most existing sleep programs follow a top-down model, training caregivers to implement interventions for youth to follow. In contrast, our interviews revealed that youth and caregivers often held divergent views on both the causes of sleep problems and the acceptability of certain strategies. These tensions around divergent views of sleep problems and strategies suggest that sustained behavior change is unlikely unless both youth and caregivers are engaged as equal contributors. Nova Sleepcare's ‘Pathway to Better Sleep’ explicitly supports pediatricians in helping families to voice their perspectives, broker compromises, and establish shared, achievable goals. Positioned as trusted medical experts, pediatricians are uniquely placed to mediate these conversations and increase the likelihood of follow-through (62–64). By anchoring goal setting in the clinical encounter, where youth and caregivers are both present, Nova Sleepcare may improve adherence and foster a greater sense of agency for the youth, a critical component in managing long-term behavioral health.

Importantly, pediatricians found Nova Sleepcare applicable across a range of patient presentations. Its content was perceived as comprehensive yet accessible, aligning with clinical guidelines while remaining adaptable to the realities of complex, co-occurring conditions. Through iterative co-design, physician impressions of Nova Sleepcare became more positive as it became more streamlined and responsive to their clinical needs. The final version offered a pragmatic reference that could be flexibly applied depending on patient and family context, time available, and the physician's own level of experience.

Limitations of this study include a geographically limited sample and English-only interviews, which may reduce generalizability. Future work will focus on how Nova Sleepcare is delivered, as developing the content is only half of the challenge; implementation strategies are where many promising programs encounter the “implementation cliff” (43). A companion paper presents the results of a consensus study designed to identify optimal implementation approaches, ensuring Nova Sleepcare can be scaled and sustained across diverse clinical settings (66).

Nova Sleepcare represents a pragmatic, innovative step toward improving sleep and reducing BoC in youth with NDDs, a population in urgent need of timely, accessible, and context-sensitive support. By embedding sleep education into the clinical encounter and offering tailored content that reflects the lived experiences of youth, caregivers, and pediatricians, Nova Sleepcare addresses a longstanding gap in pediatric care (45, 46, 65). Its co-design process ensures both usability and real-world relevance, and, together with the implementation strategies outlined in the companion paper (66), positions it as a promising intervention capable of overcoming the barriers that have limited prior programs.

## Data Availability

The original contributions presented in the study are included in the article/Supplementary Material, further inquiries can be directed to the corresponding author.
